# Prolonged morphine exposure during adolescence alters the responses of lateral paragigantocellularis neurons to naloxone in adult morphine dependent rats

**DOI:** 10.1186/s12576-021-00810-4

**Published:** 2021-08-24

**Authors:** Sara Sabuee, S. Mohammad Ahmadi-Soleimani, Hossein Azizi

**Affiliations:** 1 Department of Physiology, Faculty of Medical Sciences, Tarbiat Modares University, Tehran, Iran; 2 Deparment of Physiology, School of Paramedical Sciences, Torbat Heydariyeh University of Medical Sciences, Torbat Heydariyeh, Iran

**Keywords:** Adolescence, Morphine, Single-unit recording, LPGi nucleus, Rat

## Abstract

**Introduction:**

Adolescence is a critical period in brain development, and it is characterized by persistent maturational alterations in the function of central nervous system. In this respect, many studies show the non-medical use of opioid drugs by adolescents. Although this issue has rather widely been addressed during the last decade, cellular mechanisms through which adolescent opioid exposure may induce long-lasting effects are not duly understood. The present study examined the effect of adolescent morphine exposure on neuronal responses of lateral paragigantocellularis nucleus to naloxone in adult morphine-dependent rats.

**Methods:**

Adolescent male Wistar rats (31 days old) received increasing doses of morphine (from 2.5 to 25 mg/kg, twice daily, s.c.) for 10 days. Control subjects were injected saline with the same protocol. After a drug-free interval (20 days), animals were rendered dependent on morphine during 10 days (10 mg/kg, s.c., twice daily). Then, extracellular single-unit recording was performed to investigate neural response of LPGi to naloxone in adult morphine-dependent rats.

**Results:**

Results indicated that adolescent morphine treatment increases the number of excitatory responses to naloxone, enhances the baseline activity and alters the pattern of firing in neurons with excitatory responses in adult morphine-dependent rats. Moreover, the intensity of excitatory responses is reduced following the early life drug intake.

**Conclusion:**

It seems that prolonged opioid exposure during adolescence induces long-lasting neurobiological changes in LPGi responsiveness to future opioid withdrawal challenges.

## Introduction

Opioid derivatives have long been used in societies for both therapeutic (mainly analgesic) and euphoric (rewarding) purposes. However, long-term consumption of these drugs is accompanied with undesirable behavioral changes, i.e., tolerance and dependence. In brief, these are defined as gradual loss of drug effect following prolonged exposure and expression of withdrawal signs following cessation of drug intake, respectively [1–6]. Although the inclination for opioid abuse is not limited to a specific age, there is evidence indicating that adolescents are more susceptible to show desire for psychostimulants. This seems to be associated with increased expression of risk-taking behaviors and novelty seeking during this period [7–9]. In addition, previous studies have shown that adolescent drug abuse causes long-lasting neurobiological changes in brain function [10, 11]. In other words, the reward-related brain regions of an adult brain with the history of drug exposure during adolescence, respond differently to new drug challenges, compared to those with a clear substance background. In this regard, our previous investigation showed that rats that had undergone chronic morphine administration during adolescence, display more severe dependence and accelerated tolerance to morphine in adulthood [12]. Also, we observed that the neuronal responses of lateral paragigantocellularis (LPGi) nucleus to acute morphine injection are altered in adult rats with the experience of adolescent morphine exposure [13]. Our research works targeted the LPGi, because it is well recognized in literature as a key brain center mediating opiate effects [14–16]. For example, electrical stimulation of LPGi causes withdrawal-like signs in rats [17]. This brain structure provides the main source of excitatory inputs to the locus coeruleus (LC) nucleus [18–22]. This glutamatergic transmission results in hyperactivity of LC noradrenergic neurons and this state has been shown to temporally correspond to the expression of somatic withdrawal symptoms [23]. Interestingly, the mentioned over-activation of LC is attenuated following lesions of LPGi [24]. The functional connectivity of LPGi–LC in association with opioid withdrawal has been well demonstrated in vitro using electrophysiological experiments [19]. Recently, this reciprocal circuit between the two nuclei was comprehensively reviewed introduced to researchers as “Paragiganto-coerulear” pathway [25]. Aside from this, the LPGi projects enkephalinergic fibers to the nucleus tractus solitarius (NTS). These innervations promote the aversive features of morphine withdrawal [26].

It should be mentioned that the LPGi neuronal responses to acute morphine is electrophysiologically heterogeneous (inhibitory, excitatory and no-effect responses have been reported), and this dissimilar pattern of response makes the whole region worthy of further in-depth investigations to reveal how the intricate neural circuitry is tuned and controlled. Thus, in connection with our previous findings, the present study was designed to address an unprecedented aspect of the issue, i.e., the LPGi neuronal activity during opioid withdrawal in adult rats with the history of prolonged morphine challenge in their adolescence. The results of this research enable us to interpret how previous behavioral observations (exacerbation of opioid dependence in adulthood) could be explained at cellular levels. In addition, we aimed to clarify whether adolescent morphine exposure can differentially alter the LPGi responsiveness to prolonged (rather than acute) morphine intake in future life. Since acute doses of opioid drugs are commonly used in clinic for pain management, profound distinction of mechanism underlying both acute and chronic effects would assist the clinicians to proceed with brighter therapeutic perspective either in the context of addiction or pain management, particularly in subjects reporting pre-exposure of opioid drugs in their adolescence.

## Materials and methods

### Animals

In the present study, a total number of 73 male Wistar rats, within the age range of 23–25 days, were used (Razi Institute, Tehran, Iran). Animals were housed four per cage in a colony room with stable temperature, 12-h light/dark cycle (lights on at 7:00 a.m.) and ad libitum access to food (pellet chow) and water. Animal cages were cleaned twice a week and animals were daily checked to ensure their general health and comfort. All procedures were performed during the light hours (8 a.m.–13 p.m.) and in line with the guidelines of the ethics committee at faculty of medical sciences, Tarbiat Modares University (IR.MODARES.REC.1397.108), which corresponds to the NIH guide for the care and use of laboratory animals.

### Drugs and reagents

The following chemicals were purchased and used in our experiments: morphine sulfate powder (Temad, Tehran, Iran), naloxone hydrochloride dehydrate (Sigma-Aldrich, USA) and urethane powder (Merck, Germany). These chemicals were all dissolved in physiological saline (0.9% NaCl). Solutions were freshly prepared according to the daily need for each set of experiments. Drug injections were made through subcutaneous (s.c.) and intraperitoneal (i.p.) route for morphine and naloxone, respectively. Pontamine sky blue 2% (Sigma-Aldrich, USA) was dissolved in 0.5 M sodium acetate (Sigma-Aldrich, USA) and used as an electrolyte inside the glass micropipettes.

### Morphine administration protocols and experimental design

**Fig. 1:**
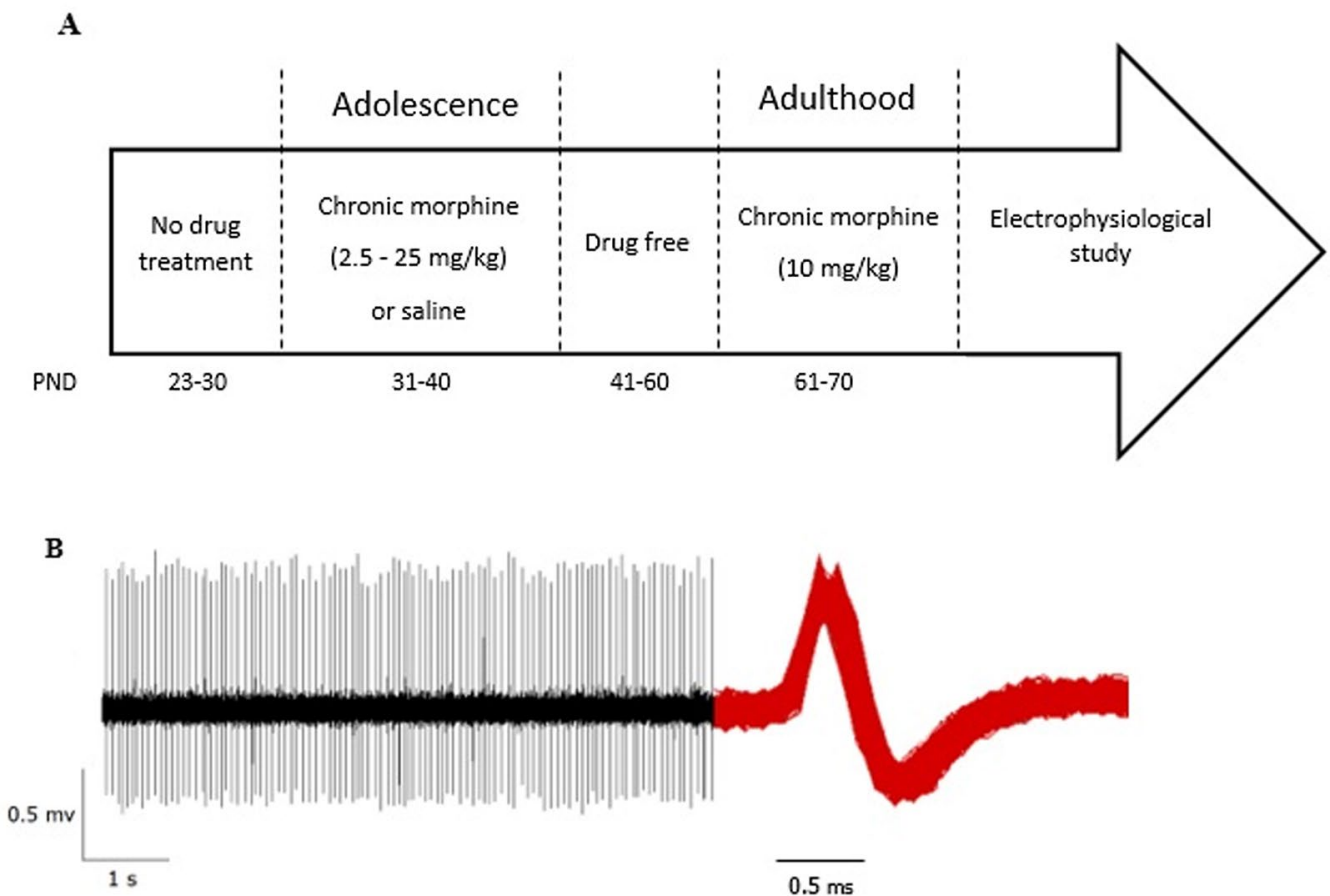
Schematic timeline indicating the sequence of experimental protocols used in this study. **A** As shown, animals receive morphine first during adolescence and then during adulthood. The periods of drug exposure are separated by a drug-free interval. *PND* post-natal day. **B** Sample of in vivo extracellular recording from LPGi region

Adolescent rats (31 days old) were randomly separated into two experimental groups to receive saline (*n* = 39) or morphine (*n* = 34) during this period. Escalating doses of morphine were administered twice daily (9:00 a.m. and 5:00 p.m.) on days 31–40, as described previously [13] and shown in the timeline (Fig. 1A). In this protocol, rats received morphine at the dose of 2.5 mg/kg on day 1 and on each subsequent day the dose was increased by 2.5 mg/kg until day 10 on which the final dose reached 25 mg/kg. Animals received no drug on days 41–60 (drug-free interval) and this period was followed by induction of morphine dependence in the same rats during adulthood (days 61–70, 10 mg/kg, twice daily, s.c.). Electrophysiological experiments were then conducted on these morphine-dependent rats with the history of adolescent morphine exposure.

### Surgical preparation and electrophysiological recording

Detailed surgical protocols are fully described in our previous studies [13, 16, 27]. In brief, adult rats (70 days old) were anesthetized by urethane injection (1.2 g/kg, i.p.). Anesthesia was stably maintained by additional doses of drug (0.15 g/kg) administered every 1 h. In order to minimize the jaw movements, rats first underwent tracheostomy and then fixed in a stereotaxic frame (Narishige, Japan) by two ear bars and an incisor piece set at − 3.3 mm. In the next step, LPGi coordinates were obtained from the rat brain atlas [28] and right above the nucleus, a tiny hole (2 mm in diameter) was drilled on the skull surface (12 mm caudal to bregma, and 1.5 mm lateral to midline). Spike activities of LPGi neurons were recorded by glass micropipettes (OD: 1.2 mm, ID: 0.94 mm, Sutter Instruments, USA) which were pulled using a horizontal programmable microelectrode puller (P-97, Sutter Instrument, USA) and filled with a solution containing 2% pontamine sky blue dye dissolved in 0.5 M sodium acetate (3–12 MΩ resistance) [13, 16]. Micropipettes were gently lowered down into the brain to reach the LPGi region (located nearly 10.2–10.8 mm ventral to the skull level). During the insertion of electrodes, a transient burst firing pattern was observed in almost all experiments indicating the proper passage of microelectrode through Bötzinger and/or pre-Bötzinger nuclei. This typical observation has previously been used as an electrophysiological landmark to ensure the methodological precision [13, 16, 27, 29]. By further insertion of electrode, the mentioned burst pattern disappears and the next emerging spike activities belong to the LPGi neurons (Fig. 1B).

Extracellular single-unit activities were amplified (× 1000) using an AC differential amplifier (DAM 80, WPI, USA). The recorded spikes were then filtered (0.3–3 kHz bandpass) and passed through an oscilloscope (Hitachi, Japan) and an audio analyzer set (Fredrick Haer, USA) for simultaneous visual and auditory monitoring. Signals were continuously digitized by a commercial analog-to-digital converter (PowerLab 4/30, AD Instruments Pty Ltd., Australia). Data were analyzed by the spike histogram module in Lab Chart 7 software (AD Instruments) and values were displayed as rate histogram (1 min bin size).

Similar to our recent study [30], electrophysiological recordings were initiated 2 h after the last morphine injection (on PND 70). First, the baseline activity of LPGi neurons was recorded for 10 min, then, naloxone injection was done (2 mg/kg, i.p.) and recording was continued for 50 min afterwards. From each rat, the spike activity of only one neuron was recorded. Any increase or decrease in spontaneous firing rate was defined as an excitatory or inhibitory response (this was calculated by mean ± 2 × standard deviation). Furthermore, the coefficient of variation (CV) for inter-spike intervals (ISIs) was obtained (SD/mean ISIs × 100) as an index of firing regularity for each neuronal response to naloxone (excitatory/inhibitory) in both experimental groups. It should be mentioned that the timing and interval of morphine/naloxone administration was fixed in all experiments.

### Statistical analysis

GraphPad Prism software (version 6.01 for Windows, USA) was used for data analysis. Data were checked for normality of distribution by the Kolmogorov–Smirnov or the Shapiro–Wilk tests. The effect of acute naloxone on mean discharge rates of LPGi neurons was analyzed by paired Student’s *t*-test, when values were normally distributed. However, Mann–Whitney *U* test was used in case data distribution was not normal. Chi-square test was used to analyze the distribution of responses. Data are presented as mean ± standard error of the mean (SEM). In all experiments, *P* < 0.05 was considered statistically significant.

## Results

### Heterogeneous responses of LPGi neurons to naloxone in adult morphine-dependent rats

**Fig. 2:**
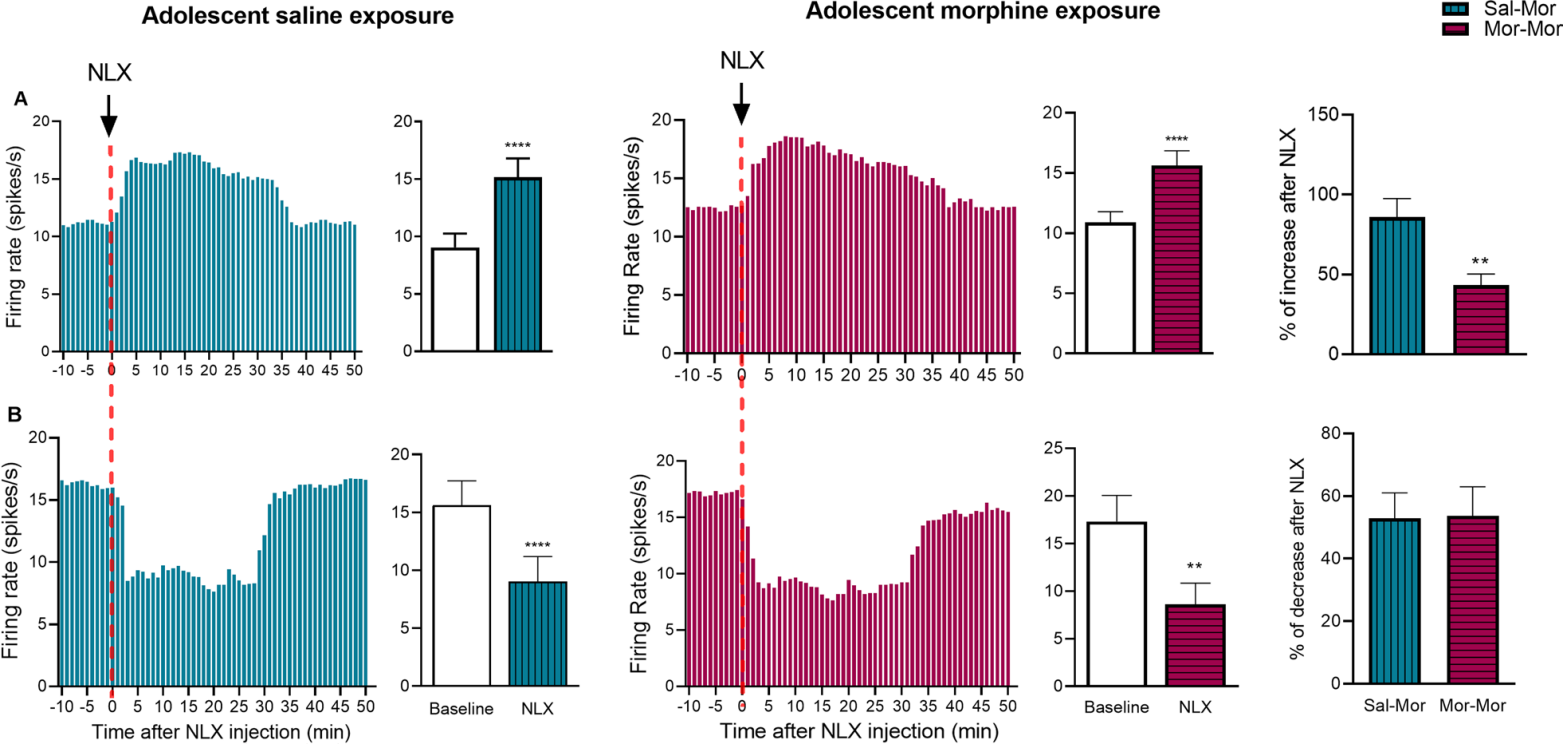
Heterogeneous responses of LPGi neurons to naloxone. As shown, naloxone injection in adult morphine-dependent rats causes excitatory (**A**) and inhibitory (**B**) responses in rats receiving either saline or morphine during adolescence. Also the intensity of excitatory responses (% of increase) is reduced in rats with history of adolescent morphine exposure. *NLX* naloxone, *Sal* saline, *Mor* morphine. ****P < 0.001, **P < 0.01. Data are expressed as ± standard error of the mean (SEM)

As indicated in Fig. 2 (histograms, parts A and B), naloxone injection in adult rats of both morphine- and saline-treated groups, resulted in differential changes of spontaneous firing which is hereafter referred to as “excitatory” and “inhibitory” responses.

### Adolescent morphine exposure alters the intensity of excitatory responses to naloxone

The intensity of responses to naloxone was calculated as the % of increase/decrease for excitatory/inhibitory responses, respectively. As illustrated in Fig. 2, the intensity of excitatory response is reduced in morphine-treated rats (43.40 vs. 85.97, *P*-value = 0.0012). However, the intensity of inhibitory responses was not affected by adolescent morphine exposure. In other words, no significant difference was observed between the two groups in this respect. In addition, all recorded signals were assessed to reveal whether morphine challenge during adolescence affects the timing of response to naloxone. This was done by calculating the onset of effect (mean ± 2SD) and time taken to reach the peak effect, regardless of the response type. Statistical analysis showed no significant difference in these parameters between the two groups (data not shown).

### Adolescent morphine exposure alters the baseline activity of neurons with excitatory response

**Fig. 3:**
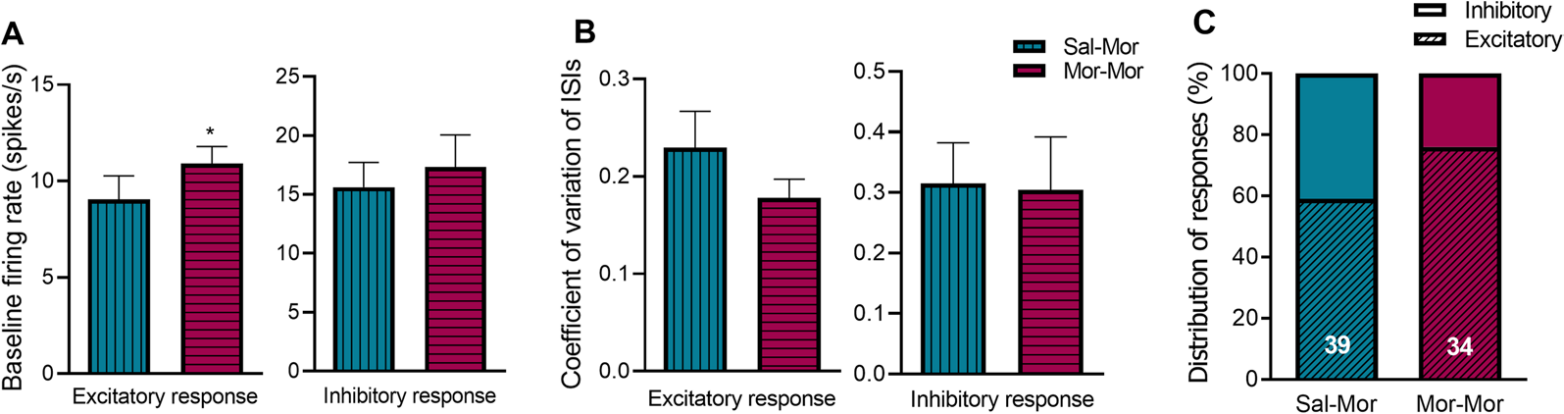
Baseline activity, coefficient of variation and distribution of responses to naloxone. The effect of adolescent morphine exposure on spontaneous firing rate (**A** for excitatory and inhibitory responses: *n* = 26 and 8, respectively). The effect of adolescent morphine exposure on CV of ISIs (**B** for excitatory and inhibitory responses: *n* = 26 and 8, respectively). The effect of adolescent morphine exposure on distribution of response types (**C** for excitatory and inhibitory responses: *n* = 26 and 8, respectively). *P < 0.05 vs. Sal–Mor group, *Sal* saline, *Mor* morphine. Data are expressed as ± standard error of the mean (SEM)

Investigation of baseline activity in all recorded signals indicated that, in morphine-treated group, neurons with excitatory response to naloxone have higher baseline firing rate compared to the control group (10.91 vs. 9.05 *P*-value = 0.045, Fig. 3A). However, in neurons with inhibitory response, no difference was found between the two groups (17.32 vs. 15.62, *P*-value = 0.638, Fig. 3A).

### Adolescent morphine exposure alters the pattern of firing in neurons with excitatory response

**Fig. 4:**
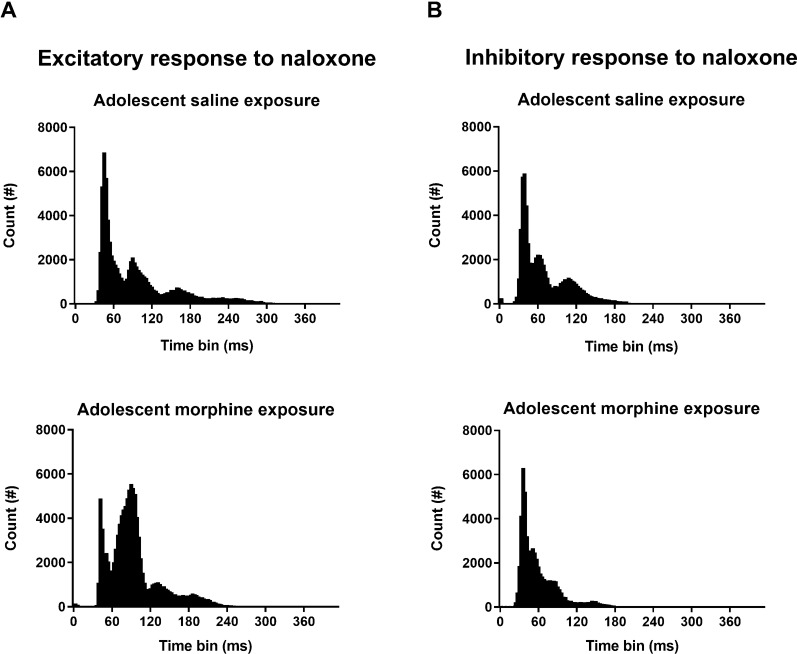
Alteration of baseline firing pattern following adolescent morphine exposure. As shown, prolonged morphine exposure during adolescence has altered the distribution of baseline ISIs in neurons with excitatory (**A**), and inhibitory (**B**) responses to naloxone (the rightward shift in peak **A** represents more ISIs with longer durations)

In order to reach an estimate of changes in firing pattern, distribution of inter-spike intervals (ISIs) was investigated. As shown in Fig. 4, in excitatory responses of saline-treated group, majority of ISIs fall within the duration around 50 ms, while, in respective responses of morphine-treated group, this pattern undergoes distortion such that most events display higher values (around 90 ms). Interestingly, when assessed among the inhibitory responses, ISI distribution profile was not remarkably affected by adolescent morphine exposure. In addition, coefficient of variation (CV) of ISIs was measured as an index of firing variability. However, no significant change was found in either of the responses between the two experimental groups (Fig. 3B).

### Adolescent morphine exposure alters the pattern of heterogeneity in response to naloxone

In order to compare the distribution of responses between the two groups, results are normalized as the percentage of occurrence for each type (Fig. 3C). As shown, adolescent morphine exposure caused more LPGi neurons to exhibit excitatory responses to naloxone in adulthood, when compared to the control group (76% vs. 59%). In morphine-treated group, total recordings (*n* = 34) included 26 excitatory vs. 8 inhibitory responses. In saline-treated animals, the heterogeneous pattern included 23 excitatory vs. 16 inhibitory responses (*n* = 39) (Fig. 3C).

## Discussion

Currently, there is evidence indicating that adolescent morphine exposure increases the severity of morphine dependence in adulthood [26]. This means that rats with the history of chronic morphine intake during adolescence display more severe withdrawal symptoms in adulthood. This effect is not merely limited to dependence, such that even development of morphine analgesic tolerance is facilitated in animals with the history of adolescent morphine intake [12]. This finding implies that the adult subject needs to more rapidly increase the dose of morphine to reach the initial experienced effect. Thus, taken together, the subject is not only at a higher risk of overdose, but also, it would be more difficult to cease drug use because of more physical suffering during the abstinence. Now, let us discuss this point more mechanistically at cellular level. As aforementioned, LPGi region is known as a significant modulator of opioid withdrawal syndrome. Thus, in the present study, we aimed to specifically reveal whether neuronal responsiveness of this nucleus is altered following induction of withdrawal state in adult rats received morphine during adolescence. The significance of this question is rooted in the pivotal role of LPGi in regulation of LC activity, as mentioned earlier. In other words, any alteration in firing rate and/or pattern of LPGi neurons would result in alteration of LC neuronal discharge which could in turn affect the expression of withdrawal behaviors. Our results indicated that naloxone injection causes heterogeneous responses in LPGi neurons of adult morphine-dependent rats with adolescent history of either saline or morphine exposure. This finding is consistent with previous reports indicating the heterogeneity of electrophysiological characterizations in the same region [13, 14]. This differential responsiveness to naloxone is rather expected regarding the extensive projections of LPGi to other brain areas and activity of various neurotransmitter systems within the nucleus [14, 25, 31, 32]. As mentioned earlier, we have previously demonstrated that adolescent morphine exposure could alter the responses of LPGi neurons to acute morphine injection in adult male rats [13]. Furthermore, development of tolerance and intensity of dependence to morphine was found to be potentiated in adulthood of rats that had undergone adolescent morphine challenge [12]. These findings motivated us to investigate how morphine-exposed LPGi neurons may electrophysiologically react to pharmacological induction of withdrawal by naloxone in adult rats that have been chronically exposed to morphine in their adolescence. First, we observed that the number of excitatory (and not inhibitory) responses is increased in morphine-treated animals compared to saline-treated ones (Fig. 3C). In other words, it seems that adolescent morphine exposure disturbs the normal distribution of responses to naloxone in LPGi region. Our further analysis revealed that, in morphine-treated rats, baseline activity of neurons with excitatory response is increased significantly compared to control animals (Fig. 3A). This brings to mind the notion that increased number of excitatory responses may, at least somehow, result from elevation of baseline activity in particular neurons. Alteration of baseline firing rate in several brain regions has previously been reported in animals that experienced prolonged amphetamine exposure during adolescence [33]. As for the opioids, there is evidence indicating that the excitatory effects of morphine on central nervous system (CNS) is primarily mediated through disinhibitory mechanisms [34–37] and this model has also been specifically suggested for LPGi region [14]. Thus, one possible explanation is that prolonged morphine intake during adolescence may increase the baseline activity of some LPGi neurons by suppression of inhibitory inputs to this region. In line with this idea, electrophysiological experiments have shown that LPGi receives GABAergic projections from nucleus tractus solitarius (NTS) and nucleus raphe magnus (NRM) [38], both of which might be affected (hyperpolarized) by morphine. However, if this was true, application of naloxone (as an opioid receptor antagonist) must have promoted the inhibition of LPGi neurons secondary to the removal of morphine disinhibition. On the contrary, in our study, naloxone injection increased the number of excitatory responses which consequently rules out the possibility of the raised hypothesis. A more rational approach might be attributable to development of cellular tolerance to morphine in LPGi neurons. This phenomenon has already been shown by in vivo experiments where application of morphine into the LPGi of morphine-dependent rats failed to alter the spontaneous firing rate [39]. With this in mind, it is possible that following long-term application of morphine in adolescence, some LPGi neurons first exhibit excitation (during the first doses) and then gradually undergo tolerance such that their firing rate reduces and remains slightly over the pre-drug baseline.

As aforementioned, the extent of naloxone-induced excitation was reduced in morphine-treated group compared to saline-treated animals; however, the intensity of inhibitory responses was not differentially affected. This raises two important points: first, prolonged morphine does not affect the LPGi neurons with the same intensity and second, the attenuated level of excitation in morphine-treated rats might be due to the increased baseline firing rate, as discussed earlier. In this regard, similar intensity of inhibitory responses is consistent with lack of significant difference in their baseline activity. Taken together, our results indicate that long-term exposure to morphine during adolescence causes neuroadaptations which ultimately result in enhancement of LPGi excitatory output following withdrawal induction. Thus, aside from the intrinsic spontaneous firing of LC, any increase in LPGi output would in turn promote the firing of LC and the subsequent potentiation of withdrawal signs.

Another aspect of our analysis addressed the pattern of spike activity in LPGi neurons of morphine-treated vs. saline-treated rats. As shown in Fig. 4, distribution of baseline ISIs was distorted in rats with history of adolescent morphine intake. ISI histogram indicates that in saline-treated group, most ISIs have a duration of less than 60 ms, while, in morphine-treated group, majority of events display durations of around 90 ms. This rightward shift of mode represents reduced probability of burst-like firing. In addition, when ISI value increases (rightward shift occurs in peaks), it means that the relative refractory period has extended which may decrease neuronal excitability to external stimuli. This interpretation is in line with reduced intensity of excitation by naloxone in morphine-treated group, as earlier mentioned. This raises the possibility of changes in conductance of potassium channels during membrane repolarization and hyperpolarization. Indeed, further studies are required to specifically investigate the proposed mechanisms at cellular level.

## Conclusion

According to our results, it seems that prolonged exposure to morphine during adolescence, as a critical phase of CNS maturation, leaves persistent changes in neuronal responses of LPGi region to naloxone. In other words, LPGi nucleus of a rat with adolescent morphine background, functions differently during withdrawal state, compared to that of a naïve subject.

## Data Availability

The datasets analyzed during the current study are available from the corresponding author on reasonable request.
